# Different Pollinators’ Functional Traits Can Explain Pollen Load in Two Solitary Oil-Collecting Bees

**DOI:** 10.3390/insects11100685

**Published:** 2020-10-11

**Authors:** Maureen Murúa

**Affiliations:** Centro GEMA: Genómica, Ecología y Medio Ambiente, Facultad de Estudios Interdisciplinarios, Universidad Mayor, Camino La Pirámide 5750, Santiago 8580745, Chile; maureen.murua@umayor.cl

**Keywords:** *Calceolaria*, *Centris*, *Chalepogenus*, Chile, functional traits, oil-collecting bees, pollen load

## Abstract

**Simple Summary:**

Pollinators’ features, especially those with a functional role during the pollination process, have been shown to be a good predictor of pollen load for some bee species, but little is known about solitary bees. Here, I have used two solitary oil-collecting bees to understand the impact of functional pollinator traits on the pollen load of two oil-secreting *Calceolaria* herb species. I therefore measured the visitation frequency, the time spent manipulating the flower and the body size and pollen load for each bee species. The results reveal that each pollinator species visits different *Calceolaria* species (*C. cavanillesii* and *C. filicaulis*) for pollen and/or oil and a few other herb species for pollen collection. In addition, each bee species showed different features involved in *Calceolaria* pollen collection and load. In the case of *C. chilensis,* only its body size affected its pollen load, while in *C. subcaeruleus*, both its body size and the time spent manipulating the flower affected the total pollen that this bee was able to carry. These results highlight the role of pollinators’ functional traits at different stages of the pollination process, specifically during pollen collection and transport, and even more in specialized plant-pollination systems.

**Abstract:**

Functional traits have been shown to be a good predictor of pollen load for some pollinator bee species, but little is known about solitary bees. In this study, I used two solitary oil-collecting bees to explore the impact of functional traits on the pollen load of two oil-secreting *Calceolaria* species. I therefore measured the visitation frequency, the time spent manipulating the flower, pollinator body size and pollen load for each bee species. The results reveal that each pollinator visits different *Calceolaria* species (*C. cavanillesii* and *C. filicaulis*) for pollen and/or oil and at least another four herb species for pollen in different proportions. In addition, each bee species presents different functional traits that affect *Calceolaria* pollen load. For *C. chilensis*, it was only its body size that affected the *Calceolaria* pollen load, while in *C. subcaeruleus,* both body size and handling time together account for its pollen load. Overall, these results highlight the role of pollinators’ functional traits in different stages of the pollination process, and even more in specialized plant-pollination systems.

## 1. Introduction

Pollination is a complex process whose success depends on the traits of both the plant and its pollinators. Historically, greater importance has been placed on establishing how the arrangement of different floral traits could make up pollination syndromes mediated by the taxonomic and morphological diversity of certain pollinator species [1,2,3,4,5,6], and the role of functional diversity has tended to be overlooked. The way in which pollinators interact with their flowers [7] may often be of greater importance than the number of species that visit a certain plant. In fact, different aspects of functional pollinators’ traits, such as the visitation frequency, the time spent in the flower and pollinator body size, may together affect floral resource exploitation and therefore the pollination of a given plant species.

In general, it is expected that an increase in the visitation frequency to a given plant may favor greater pollen extraction or that pollinators with greater body size can carry larger pollen loads [8]. To take honeybees as an example, it has been observed that large foragers carry heavier loads, increasing their total mass by up to 40% [9]. However, visit frequency or total body size cannot totally explain pollen load size, since larger pollinator species often make brief visits, decreasing the probability of pollen collection; thus, in such cases, other features could be more relevant to the amount of pollen extracted. For example, a study of *Eurysimum mediohispanicum* populations [10] found that large bees make short visits, but they handle the flower very quickly and with great accuracy, thereby increasing their total pollen load. In this sense, the pollinators’ handling abilities could be a key factor in improving the amount of pollen extracted per visit, since staying longer can increase the probability of them extracting more grains per visit [11]. 

Obviously, plants are not isolated in the landscape, but rather inserted into a wide community of other floral species that are constantly competing for pollinators [12]. In this context, and according to their nutritional requirements, pollinators will often visit several plant species during one foraging trip and carry a mixture of pollen grains [13], and this will have different consequences to plant reproduction [14]. In addition, if we consider that individuals could have a maximum pollen load capacity [15] related to their body size, the way in which the insect interacts with the flower could be critical to ensuring appropriate conspecific pollen collection. It is therefore thought that not just one but rather a combination of several functional pollinators’ traits will determine the specific amount of pollen that a certain insect can carry and potentially deposit. 

While these aspects can potentially affect all plant species, they can be particularly critical for more specialized ones, which depend on a limited number of pollinators for fertilization. Sometimes, this type of specialized relationship can be asymmetrical, where a pollinator will visit many different plant species in the landscape, but a particular plant depends on just one or a few species for its reproduction [16]. In such cases, the pollinators’ functional features will be a key determinant of the effective conspecific pollen transfer and deposition. This is the case with oil-collecting bees and oil-secreting flowers, a highly specialized plant–pollinator relationship that involves only eleven families of Angiosperm and fourteen genera of bees [17,18,19,20]. Most of these bees are distributed throughout South America. In Chile, there are at least two genera of solitary bees (i.e., *Centris* and *Chalepogenus*, both belonging to Apidae family) that collect oil as a floral reward from different species of the oil-secreting *Calceolaria* genus (Calceolariaceae) [21,22,23]. 

The *Centris* genus is composed of 200 species, where most of the females have a special structure, an oil collector, consisting of two combs located on the ventral surface of the basitarsus [19,24]. The *Centris* species flies quickly and this enables them to travel long distances, following fixed routes and making short visits to most of the flowers present in an inflorescence [25]. During the oil collection, the individual clutches the flower with their midlegs and collects the oil with their forelegs by making sweeping movements with them. Then, during flight, it passes the oil from its forelegs to its hindlegs with the help of its midlegs [25]. Meanwhile, the *Chalepogenus* genus comprises 21 species with oil collectors only on their forelegs. Their oil-collectors take the form of a pad of finely branched hairs, located on the posterior surface of the forebasitarsus [26]. During the foraging process, they visit small plant populations and try to visit all the available flowers, even returning to the same patch more than once. When they are collecting oil, these bees perform a tap movement, where the oil is absorbed by capillarity [24]. Once the oil has been extracted from the flower elaiophore, it is transferred from the forelegs to the hindlegs without the bee leaving the flower [25]. 

*Calceolaria* is a diverse American genus, whose evolution is hypothesized to have occurred in close relationship with its pollinators. However many of the mechanisms behind this process are still unknown. In Chile, it is one of the four most diversified genera of plant species [27] and recent research has described floral syndromes associated with different genera of oil-collecting bees. Nonetheless, plant–pollinator functional trait associations and their impact on different steps of the pollination process remain hardly explored. To overcome part of this current gap, I have used two species of oil-collecting bees belonging to *Centris* and *Chalepogenus* to study the differential impact of pollinators’ functional traits on pure *Calceolaria* pollen load. Specifically, I determined how functional traits, such as visitation frequency, time spent manipulating the flower and pollinator body size can affect the size of *Calceolaria* pollen load. While both pollinator species specialize in collecting oil from the *Calceolaria* genus, the behavioral differences described above during reward collection suggest that different combinations of functional traits could affect pure *Calceolaria* pollen load.

## 2. Materials and Methods 

### 2.1. Study Site and Plant–Pollinator Species

**Figure 1:**
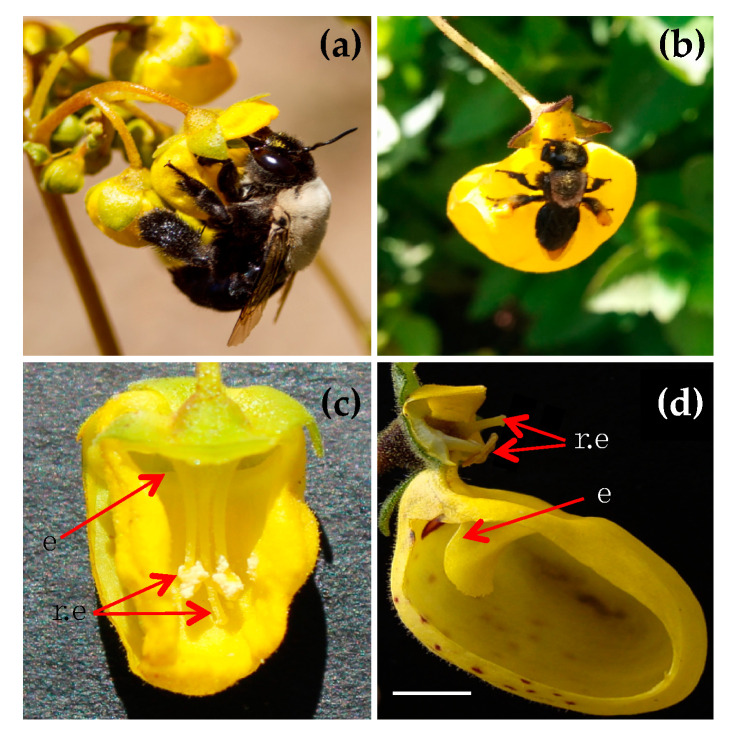
Oil-collecting bees and oil-secreting flowers. (**a**) *Calceolaria cavanillesii* and *Centris chilensis*, (**b**) *Calceolaria filicaulis* and *Chalepogenus subcaeruleus*, (**c**) aerial view of the lower lobe of *C. cavanillesii* and (**d**) lateral view of *C. filicaulis*. The arrows indicate the position of the reproductive structure (r.e) and elaiophore (e) in plant each species. The white bar represents the scale of 0.3 mm.

In January of 2019, I quantified the total pollen load and different pollinators’ functional traits of two oil-collecting bees that coexist in sympatry in the Andean mountain area of the Maule region (35°59′55.7″ S 70°33′51.6″ W) in Chile. The study site was a creek situated at 2400 masl, which has a Mediterranean-type climate with rainfall during wintertime [28]. The creek is an Andean habitat with a diverse herbal community containing species such as *Adesmia emarginata*, *Hordeum comosum* and *Rumex acetocella* and mainly dominated by two species of *Calceolaria: C. filicaulis* and *C. cavanillesii*. Along the creek, the *Calceolaria* species are exclusively pollinated by just two oil-collecting pollinator species: *Centris chilensis* (Spinola) and *Chalepogenus subcaeruleus* (Roig-Alsina). (Figure 1). However, they also visit other co-flowering species for pollen (Murúa per obs.). *Calceolaria* is a bi-labiate flower composed of an upper lobe that generally covers the reproductive structure, and a lower lobe, which contains a trichomes gland that secretes non-volatile oil as a floral reward (i.e., elaiophore; [25]). Both *C. cavanillesii* and *C. filicaulis* specifically present a yellow corolla with an elaiophore located in the same position, at the entrance to the lower lobe (Figure 1). However, the position of their reproductive structure differs between species (Figure 1a,d). *C. cavanillesii* has a sternotribic flower, so the style and stamens rest in the lower lobe and are covered by the upper lobe, which causes the pollen to be deposited in the ventral part of the bee’s abdomen during pollination (Figure 1c). Meanwhile, *C. filicaulis* has a nototribic flower, where both reproductive structures are enclosed in the upper lobe and pollen is deposited in the dorsal part of the pollinator’s head (Figure 1d). 

### 2.2. Visitation Frequency and Handling Time

During January 2019, I estimated the visitation frequency (VF) and flower-handling time (HT) of each oil-collecting bee on both *Calceolaria* species. To achieve this, I undertook a pollinator census between 9:00 and 18:00 h over a period of thirty consecutive days during the flowering season of these species. Pollinator activity was recorded only on sunny days, as the number of visits per flower that took place in a 15-min period (n◦ visits×flower^−1^×h^−1^), where only those species that contacted the plants’ reproductive structures were considered to be legitimate pollinators. In addition, I also recorded the time that each pollinator spent manipulating the corolla flower while foraging (i.e., HT), which was recorded as seconds per flower. 

### 2.3. Floral Communities, Pollinators’ Pollen Load and Body Size

In order to properly identify plant species from each pollinator’s pollen load, I developed a pollen reference library from fresh samples collected at the beginning of the sampling period (i.e., early January). To do so, I established five independent 50-m transects (total = 250 m) along the creek and every five meters I recorded the abundance and identity of all the blooming species in a 2 m^2^ quadrant. In each quadrant, I selected three floral buds per species and stored them in a 70% ethanol solution. Then, pollen grains from each plant species were photographed and characterized according to their size and shape using a microscope at 100× magnification. When pollen grains were difficult to characterize, they were pre-treated with an acid solution following the protocol in [29]. 

Every day during the 30-day period at the end of the foraging activity, I captured two or three specimens of each pollinator species using an entomological net. Once captured, I removed their third pair of legs and the rest of the body I immediately stored in Eppendorf tubes with 1 mL of ethanol at 70%. In the laboratory, I extracted all the pollen grains from each pollinator’s body by shaking the tubes with a vortex machine for 1 min. In the next step, I extracted each bee from the tube and I took ten subsamples of 3 uL per tube and put it under the microscope at 100× magnification. Then, using the reference pollen library, I counted and classified all the pollen grains that belonged to *Calceolaria* or other plant species and estimated the total amount of pollen grain by extrapolating the subsamples to 1 mL. In addition, I measured the body size (BS) of each individual, taking the total length (mm) from the front of its head to the end of the abdomen from photographs using ImageJ software (available at: https://imagej.nih.gov/). 

Finally, I estimated both the total *Calceolaria* pollen load (CP) and the pollen load for other species (OP), expressed as a total number of pollen grains per individual [30] and I used this data to determine the proportion of pure *Calceolaria* pollen load (PP) carried by each bee species (i.e., PP = CP/OP).

### 2.4. Data Analysis

To estimate statistical differences in pollinators’ functional traits (VR, HT and BS) between bee species, I performed three independent Generalized Linear Model (GLM) tests with a Gaussian error structure, using pollinators’ traits as a response variable and bee species as a factor. 

In order to determine the impact of functional pollinators’ traits on pure *Calceolaria* pollen load (PP), I fitted two different GLMs, one for each pollinator species, using a Gaussian error structure implemented with ‘stats’ package in R studio software version 1.1.453 [31]. The PP was used as a response variable and VF, HT and BS as fixed factors. Then, the models with the lowest AIC and the highest R^2^ were selected. Prior to the GLM analyses, the assumptions of normality and homoscedasticity were checked for each data set using Shapiro and Levene tests, respectively. 

## 3. Results 

### 3.1. Visitation Frequency and Handling Time

**Figure 2:**
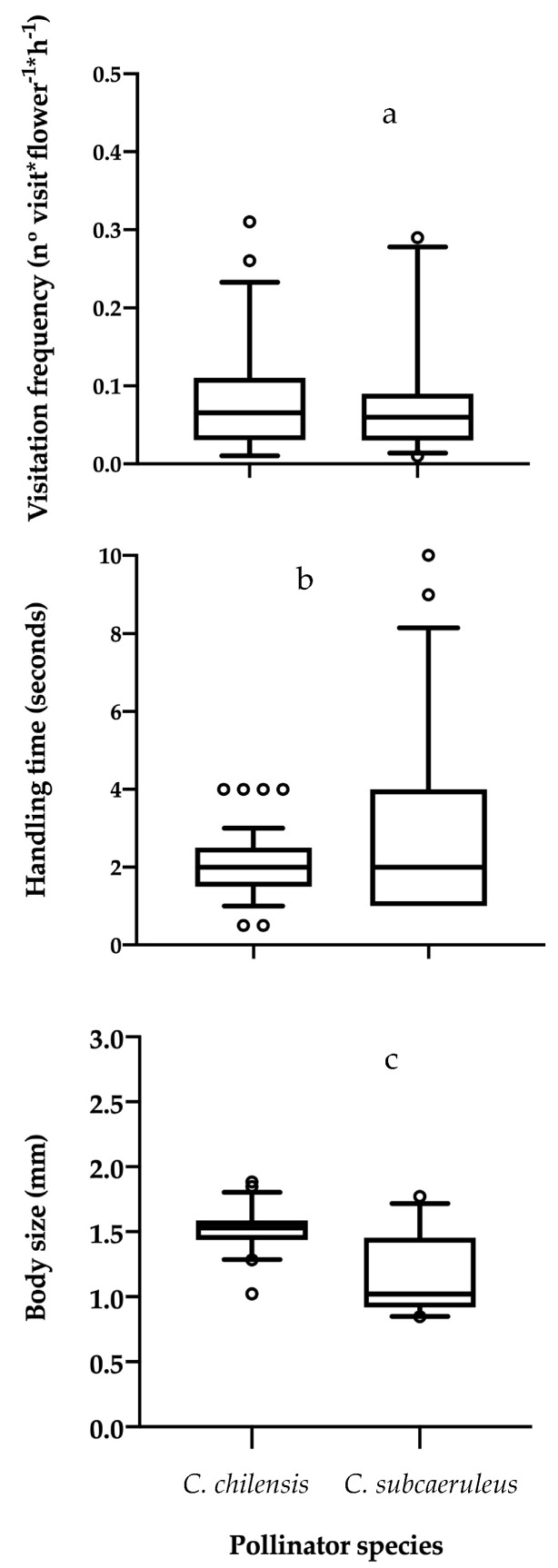
Pollinators’ functional traits measured in two oil-collecting bees. (**a**) Visitation frequency, (**b**) handling time and (**c**) body size. All values represent mean ± standard error.

During the 30-day sampling period, I spent 234 h observing *C. filicaulis* and 380 h observing *C. cavanillesii*. This observation revealed that plant species were visited by different oil-collecting bees. *C. filicaulis* was exclusively pollinated by *C. subcaeruleus*, while *C. cavanillesii* was only pollinated by *C. chilensis*, both with similar mean visitation frequencies, which did not differ statistically (GLM: t-value = −0.001, *p* = 0.99; Figure 2a). In terms of the time spent manipulating the flowers while foraging, on average *C. subcaeruleus* spent one second longer than *C. chilensis*, and these differences were statistically significant (GLM: t-value = 2.99, *p* = 0.004; Figure 2b).

### 3.2. Floral Communities, Pollinator’s Pollen Load and Body Size

**Figure 3:**
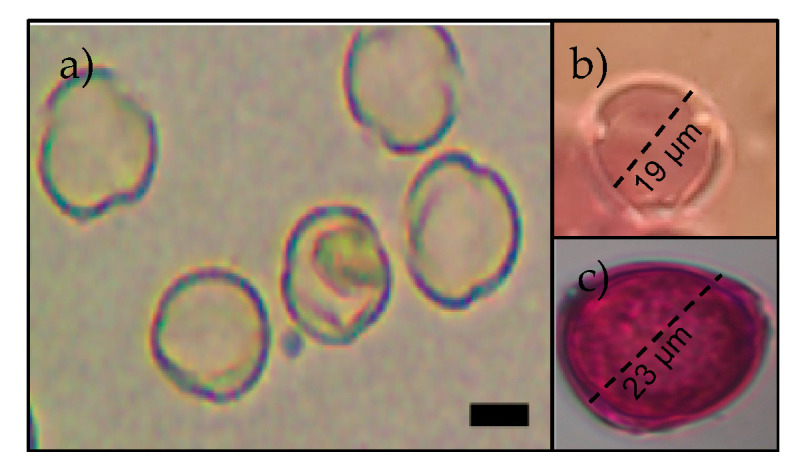
Details of a *Calceolaria* pollen grain with and without stain. (**a**) General view of pollen grains without stain, (**b**) pollen of *C. cavanillesii* and, and (**c**) pollen of *C. filicaulis*. The black bar represents a scale of 10 µm.

Along the length of the creek, 59 plant species belonging to 25 families were identified, of which *A. emarginarta* and *C. cavanillesii* were the most abundant, covering 32.2% of the sampled transect area. The laboratory analysis showed that the species presented a wide variation in pollen size, ranging from around 13 to 70 um, with *A. emarginata* having the lowest pollen grain size (13 µm) and *Collonia* sp. The biggest (60.95 µm). Both the *Calceolaria* species have similar pollen morphologies but different grain sizes, where *C. cavanillesii* has a pollen grain that ranges from 18 to 21 µm, whereas, for *C. filicaulis*, the pollen grain ranges from 22 to 23 µm (Figure 3).

During the sampling, I captured 35 individuals of *C. subcaeruleus* and 50 individuals of *C. chilensis.* On average, *C. chilensis* showed a total body size almost twice the size of that of *C. subcaeruleus*, a difference that was statistically significant (GLM: t-value= −7.73, *p* < 0.001; Figure 2c). In terms of total pollen load, *C. subcaeuleus* carried 224534 pollen grains and *C. chilensis* 6388033 pollen grains, and the pollen species composition was similar in both bees with the exception of the *Calceolaria* species (Table 1). In particular, for *C. subcaeruleus,* 42% of the total pollen load was purely *C. filicaulis*, while 60% of the pollen load of *C. chilensis* corresponded to *C. cavanillesii*. The other pollen grains were from the same plant species but in different proportions (Table 1). For example, in *C. subcaeruleus,* the second biggest contributor to total pollen load was *Phacelia* sp. (39.4%; Table 1), while, for *C. chilensis*, the second most represented species was *Plantago* sp. (25.8%; Table 1). 

Finally, for both pollinator species, the GLM model showed that different combinations of functional pollinators’ traits had a significant effect on pure *Calceolaria* pollen load (Table 2). In the case of *C. chilensis*, the model with the highest fit (AIC = −40.09; R^2^ = 0.2) included only body size as a significant statistical effect on pure *Calceolaria* pollen load (Table 2). Meanwhile, for *C. subcaeruleus,* the model with highest fit (AIC = −66.23; R^2^ = 0.56) detected significant effects on both body size and handling time and in the interaction terms between the two variables (i.e., BSxHT; Table 2). That is to say that body size and handling time together affect the amount of *Calceolaria* pollen carried by this pollinator.

## 4. Discussion and Conclusions

The aim of this work was to determine the impact of the functional traits of two oil-collecting bees on pure *Calceolaria* pollen load. Overall, the analysis revealed that both pollinators carried pollen from at least five different herb species. The pollen was present in different proportions, but *Calceolaria* was the species with the highest percentage of representation. In addition, both pollinator species presented different functional traits that affected *Calceolaria* pollen load. In *C. chilensis* only body size affected pollen load, while both body size and handling time affected pollen load in *C. subcaeruleus*.

Most of the bee species depend on flowers to acquire the necessary nutrients for their development and reproduction [32]. In the field, adults forage, collecting a mixture of pollen and nectar from different plant species to obtain the carbohydrates, proteins and lipids necessary for these functions [13]. This information has mainly been documented for different social bees of the *Apis* and *Bombus* genera [33,34], but little is known about solitary bees (but see [35]). In this study, we observed that both the *Centris* and *Chalepogenus* species forage mainly on at least four other herb species in addition to *Calceolaria*, but in different proportions. Of these species, *Calceolaria* is the only oil-secreting flower, so it is not surprising that oil-collecting bees must visit other plant species for pollen and nectar. As has been mentioned, both types of oil-collecting bee carried different proportions of non-*Calceolaria* species, which may be explained by their different energy requirements because of their different body sizes, flying distances, breeding sizes, and other factors. Unfortunately, I do not currently have access to studies that provide information about the nutritional necessities of these pollinator species, but future researchers ought to consider this aspect in order to better understand the mechanism behind pollinator preferences. 

With respect to pure *Calceolaria* pollen load, each type of bee exclusively pollinates one of the two *Calceolaria* species available at the study site, and, during over 200 h of observation, them switching between *Calceolaria* species has not been observed. The analysis of pollen load revealed that 60% of the pollen collected by *C. chilensis* corresponded to *C. cavanillesii*, while *C. filicaulis* accounted for just 42% of the pollen load of *C. subcaeruleus*. There are at least three factors that may account for these differences. First, *C. cavanillesii* showed as a bigger floral display (~ 50 flowers per plant) than *C. filicaulis* (~ 4–5 flowers per plant), and *C. chilensis,* unlike *C. subcaeruleus*, visits many flowers in one single trip, probably collecting a greater amount of pollen. Second, *Calceolaria* species have different flower types (i.e., *C. chilensis* = sternotribic flower; *C. filicaulis* = nototribic flower), which can affect the place where pollen is deposited and its retention in the pollinator’s body. It has been documented that plant species deposit pollen in “safe places” on the pollinator’s body, where it cannot be easily removed [36,37,38]. Accordingly, in *C. chilensis,* pollen is deposited on the ventral surface, where it is more difficult to groom, whereas, in *C. subcaeruleus,* most of the pollen is deposited at the front part of its head, where it is constantly groomed by its two first pairs of legs. Third, plant species show differences in the separation between flower lobes (i.e., open or closed), which may condition the access to flower rewards and affect pollen extraction. When *C. chilensis* reaches the flower, it must hang from the lower lobe in order to separate it from the upper one and so provide access to the oil. When it does this, it exposes the reproductive structure, which vibrates during oil extraction, releasing large amounts of pollen. *C. subcaeruleus* also lands on the lower lobe, but the lobes are far enough apart to easily permit access to the oil, which it achieves by making immediate contact with the stamens situated at the border of the upper lobe, which deposit a small amount of pollen on the front part of its head. The morphological relationship between corolla shape and pollinator accessibility has been previously examined and discussed for different *Calceolaria* species [4,25,39], and it has been suggested that flower structures could have a role in pollinators’ specificity and possibly in pollen deposition as well. However, to date the relationship between flower morphology and pollen collection by different pollinator species has not been quantified, so this still needs to be evaluated in depth.

Pollinator species have a set of functional traits that together facilitate pollen extraction and deposition, favoring the pollination process [40]. In this respect, the time a species takes and the efficiency with which it removes the pollen, the duration of the visit, the pollinator’s body size and even their level of hairiness can be responsible for successful pollination [41,42,43]. Here, only pollinator body size and handling time seem to play a key role in *Calceolaria* pollen load, but in different ways according to their main pollinator species. For *C. chilensis*, only its body size showed an effect on pure *Calceolaria* pollen load, whereas, for *C. subcaeruleus*, both body size and handling time accounted for pollen load. On average, *C. chilensis* has a body size that is almost twice that of *C. subcaeruleus*, which could favor more pollen being carried by *Centris*. These results are supported by other research that showed a positive relationship between body size and pollen load in some bee species [9,15], but see [44]. In the case of *C. subcaeruleus*, body size alone could not totally explain the pollen load. Their small body size may not be able to collect large amounts of pollen, but increasing the flower handling time could have a compensatory effect, possible explaining the joint effect of both functional traits on pollen load. Indeed, it was possible to observe that *C. subcaeruleus* stays longer on the flower than *C. chilensis,* since it takes the time to collect both pollen and oil during a single visit, whilst *C. chilensis* makes a short visit just to collect oil and the pollen is passively attached in its abdomen. This behavior has been described for a few species of *Chalepogenus* visiting other oil-secreting plant species [25,45], but there is scant information for many other species, especially in Chile.

Functional diversity has been recognized as a key component of diversity [46,47]. In this respect, functional traits are good predictors of total pollen load [7] and pollination efficiency [48,49]. Unfortunately, most of the evidence is based on bees inhabiting crop systems and little is known about the role of functional traits of non-commercial bees, and still less about solitary wild bees. Past research has previously revealed a relationship between *Calceolaria* morphology and pollinator specificity. However, the relationship between insect behavior and pollen collection had not been addressed until now in this first attempt to quantitatively test it. This is particularly important in specialized plant–pollinator systems, such as *Calceolaria* and its oil-collecting bees, where knowing the structural relationships between plants and insects can be useful to understand how these relationships rise, develop and evolve. Future research should include other functional traits not measured here, such as pollinator hairiness, buzzing and pollinator constancy, in order to fully understand the behavioral impact of bee species on the pollination of these specialized plant species. 

## Figures and Tables

**Table 1:** Pollen load composition of the two oil-collecting bees under study.

Pollinator Species	*Calceolaria* sp.	*A. emarginata*	*Plantago* sp.	*Phacelia* sp.	Others *
*C. chilensis*	2705 ± 204 (59.9%)	1092 ± 106 (1.6%)	44 ± 20.40 (25.8%)	2528 ± 201 (11.7%)	47 ± 13 (0.9%)
*C. subcaeruleus*	76,629 ± 7200 (42.2%)	1992 ± 380 (17%)	32,945 ± 4171 (0.7%)	15,007 ± 1372 (39.4%)	1180 ± 195 (0.7%)

The values represent mean ± standard error. The percentage of contribution of each plant species to total pollen load are reveled in parenthesis. * Unidentified species.

**Table 2:** Generalized lineal model of the effect of pollinators’ functional traits on pure *Calceolaria* pollen load for both study species. VF = visitation frequency; HT = handling time; BS = body size. Asterisks represent statistical significance.

Pollinator Species	Source	Estimate	t-Value	P
*C. chilensis*	VF	−1.332	−0.405	0.687
	HT	0.034	0.515	0.609
	BS	−0.519	−1.975	0.055 *
	VF × HT	−0.075	−0.115	0.908
	VF × BS	0.811	0.33	0.743
*C. subcaeruleus*	VF	0.804	0.881	0.389
	HT	0.108	3.382	<0.001 *
	BS	0.511	4.292	<0.001 *
	VF × HT	0.089	0.436	0.667
	VF × BS	−1.129	−1.108	0.281
	HT × BS	−0.106	−3.754	<0.001 *
